# Influenza in Children

**Published:** 1901-01-05

**Authors:** 


### INFLUENZA IN CHILDREN.

Writing on the subject of influenza in children Dr. Jacobi (New York *Medical News*) points out the measures which may be employed in the hope of keeping off the infection, but says that there is no other infectious disease of equal communicability either direct or indirect, and that only under extraordinary circumstances is there a possibility of avoiding contact. Although, however, such measures as quarantine may be of theoretical rather than of practical utility, there are certain measures which are of use in limiting the spread of infection. Thus, both sick and well children should use disinfectant mouth-washes of water slightly acidulated with hydrochloric acid. It is well also that the drinking water should be acidulated, and that irrigation of the nose so as to remove all accumulations of mucus should be a matter of course with all. As for treatment there is no specific, so that rational, hygienic, symptomatic and sustaining treatment must be trusted to. A purgative dose of calomel should be given, and the patient should be kept in bed. The diet should be scanty and fluid at first, and later, eggs, and perhaps, in a few selected instances only, alcohol and other medicinal stimulants may be given. It is, however, in the slow convalescence rather than in the acute phases of the disease that stimulants are required.

Even if there be a high temperature cold water is not indicated either as a bath or as a pack. On the other hand a warm bath is often of service when there is much muscular pain and restlessness. Hot baths, however, are to be avoided. Influenza pneumonias do better with warm packs than with cold ones. Of antipyretic drugs phenacetin is the only one of which Dr. Jacobi speaks favourably. He strongly objects to the use of acetanilid, which he regards as poisonous. There is hardly any other disease which has such a tendency as influenza to produce exhaustion and heart failure, so that stimulants may be required. These should rarely be alcoholic. Caffein preparations or strychnine are preferable. One of the best of stimulants is Siberian musk, which is useful in the gravest cases—those attended with heart failure and collapse. "Musk," he says, "together with large, hot enemata, has led me over many a difficult pass, and I again offer this experience of mine, which now extends over fifty years, as a contribution to your aid in dire distress," and not only in influenza but in all conditions of nerve exhaustion.

